# Loss of *p53* Attenuates the Contribution of *IL-6* Deletion on Suppressed Tumor Progression and Extended Survival in *Kras*-Driven Murine Lung Cancer

**DOI:** 10.1371/journal.pone.0080885

**Published:** 2013-11-15

**Authors:** Xiaohong Tan, Julian Carretero, Zhao Chen, Jishuai Zhang, Yanxiao Wang, Jicheng Chen, Xiubin Li, Hui Ye, Chuanhao Tang, Xuan Cheng, Ning Hou, Xiao Yang, Kwok-Kin Wong

**Affiliations:** 1 State Key Laboratory of Proteomics, Genetic Laboratory of Development and Diseases, Institute of Biotechnology, Beijing, China; 2 Department of Medical Oncology, Dana-Farber Cancer Institute, Boston, Massachusetts, United States of America; 3 Department of Medicine, Harvard Medical School, Boston, Massachusetts, United States of America; 4 Model Organism Division, E-institutes of Shanghai Universities, Shanghai JiaoTong University, Shanghai, China; 5 Department of Lung Cancer, Affiliated Hospital of Academy of Military Medical Sciences, Beijing, China; 6 Department of Medicine, Brigham and Women’s Hospital, Harvard Medical School, Boston, Massachusetts, United States of America; 7 Ludwig Center at Dana-Farber/Harvard Cancer Center, Boston, Massachusetts, United States of America; 8 Belfer Institute for Applied Cancer Science, Boston, Massachusetts, United States of America; 9 Department of Physiology, Faculty of Medicine and Odontology, University of Valencia, Valencia, Spain; University of Melbourne, Australia

## Abstract

Interleukin-6 (IL-6) is involved in lung cancer tumorigenesis, tumor progression, metastasis, and drug resistance. Previous studies show that blockade of IL-6 signaling can inhibit tumor growth and increase drug sensitivity in mouse models. Clinical trials in non-small cell lung cancer (NSCLC) reveal that IL-6 targeted therapy relieves NSCLC-related anemia and cachexia, although other clinical effects require further study. We crossed *IL-6* ^*-/-*^ mice with *Kras* ^*G12D*^ mutant mice, which develop lung tumors after activation of mutant *Kras* ^*G12D*^, to investigate whether IL-6 inhibition contributes to tumor progression and survival time *in vivo*. *Kras* ^*G12D*^; *IL-6* ^-/-^ mice exhibited increased tumorigenesis, but slower tumor growth and longer survival, than *Kras* ^*G12D*^ mice. Further, in order to investigate whether *IL-6* deletion contributes to suppression of lung cancer metastasis, we generated *Kras* ^*G12D*^; *p53* ^*flox/flox*^; *IL-6* ^-/-^ mice, which developed lung cancer with a trend for reduced metastases and longer survival than *Kras* ^*G12D*^; *p53* ^*flox/flox*^ mice. Tumors from *Kras* ^*G12D*^; *IL-6* ^-/-^ mice showed increased expression of TNFα and decreased expression of CCL-19, CCL-20 and phosphorylated STAT3 (pSTAT3) than *Kras* ^*G12D*^ mice; however, these changes were not present between tumors from *Kras* ^*G12D*^; *p53* ^*flox/flox*^; *IL-6* ^-/-^ and *Kras* ^*G12D*^; *p53* ^*flox/flox*^ mice. Upregulation of pSTAT3 and phosphorylated AKT (pAKT) were observed in *Kras* ^*G12D*^ tumors with *p53* deletion. Taken together, these results indicate that *IL-6* deletion accelerates tumorigenesis but delays tumor progression and prolongs survival time in a *Kras*-driven mouse model of lung cancer. However, these effects can be attenuated by *p53* deletion.

## Introduction

Accumulating evidence indicates that inflammation contributes to tumorigenesis, tumor progression, and metastasis [1,2]. Oncogene-associated inflammation leads to production of inflammatory cytokines such as interleukin-6 (IL-6) [3,4], a pleiotropic cytokine involved in inflammation, immunity, bone metabolism, neural development, reproduction, and hematopoiesis [5]. However, IL-6 is also associated with increased risk of lung cancer [6-8]. IL-6 can be detected in breath condensate of patients with non-small cell lung cancer (NSCLC) [9], and in serum of some lung cancer patients, but is not detectable in patients with benign lung disease [10]. Elevated IL-6 levels contribute to malignant pleural effusion [11,12], postoperative complications [13], and postoperative recurrence [14] of lung cancer. Several studies have correlated high circulating IL-6 levels with poor survival of lung cancer patients [15-23]. IL-6-mediated inflammation correlates with debilitating cancer-related symptoms such as fatigue, thromboembolism, cachexia, and anemia [3], and IL-6 signaling activation correlates with lung cancer chemotherapy resistance [16,24]. These studies suggest an important role for IL-6 in several aspects of lung cancer.

IL-6 expression can be detected in lung tumors [25] and in 53% of lung cancer cell lines [26], and IL-6 pathways are activated in a human lung cancer stem cell line [27-29]. Functional assays suggest that IL-6 influences the ability of cancer cells to metastasize to distant sites [30,31] and that IL-6 promotes tumor growth in a paracrine fashion *in vivo* [4,26,32]. Therefore, it is perhaps not surprising that *IL-6* knockdown, genetic ablation, or treatment with a neutralizing IL-6 antibody inhibits tumor growth *in vivo* [4,33]. Conversely, activation of IL-6 signaling contributes to resistance to epidermal growth factor receptor (EGFR) inhibitors in a mouse model of NSCLC [34,35], while blockade increases drug sensitivity in xenograft models [34]. 

An IL-6 monoclonal antibody therapy would be predicted to inhibit the inflammatory microenvironment in lung cancer. One such therapy, ALD518, has undergone preclinical and Phase I and II clinical trials. It appears to be well tolerated and ameliorates NSCLC-related anemia and cachexia [3], although the totality of clinical outcomes needs further study. 

To assess the contribution of IL-6 signaling inhibition on tumor progression and survival time *in vivo*, we crossed *IL-6* ^*-/-*^ mice with mutant *Kras* ^*G12D*^ mice because IL-6 is a downstream effector of oncogenic Ras to promote tumorigenesis[4]. NSCLC is often diagnosed with metastasis and has a poor prognosis. The treatment and prevention of lung cancer metastases are major unmet needs [36]. Inactivating mutations in *p53* are found in at least 50% of NSCLC cases [36], and *Kras* ^*G12D*^ activation accompanied by *p53* deletion can cause lung tumor metastasis [37]. To study the function of IL-6 in metastasis, we also generated *Kras* ^*G12D*^; *p53* ^*flox/flox*^; *IL-6* ^-/-^ mice..

## Materials and Methods

### Mice

*IL-6* ^*-/-*^ mice were purchased from The Jackson Laboratory and maintained in sterile housing [38]. Conditional *Lox–Stop–Lox Kras* ^*G12D*^ (hereafter referred to as *Kras* ^*G12D*^) mice [39] and *p53* ^*flox/flox*^ mice [40] were described previously. *Kras* ^*G12D*^ and *Kras* ^*G12D*^; *lL-6* ^*-/-*^ mice were inoculated with 5 × 10^6^ PFU of adenoviral Cre (adeno-Cre) by intranasal inhalation to activate oncogenic *Kras* ^*G12D*^ in the lungs. *Kras* ^*G12D*^; *p53* ^*flox/flox*^ and *Kras* ^*G12D*^; *p53* ^*flox/flox*^;*IL-6* ^*-/-*^ mice were inoculated with 5 × 10^5^ PFU of adeno-Cre. All experimental procedures were performed in accordance with the National Institutes of Health Guide for the Care and Use of Laboratory Animals. The protocol was approved by the Institutional Animal Care and Use Committee at Dana-Farber Cancer Institute (permit number 04-094). All surgeries were performed under Avertin anesthesia to minimize suffering. After euthanasia, organs, including heart, liver, spleen, kidney, stomach, intestine, spine, brain, breast, skin, and testis or ovary, were undergone gross inspection for metastases. Lung tumors adhered to the pleura were considered parietal pleural metastases. Suspected metastases were harvested and confirmed by histological features.

### Histology and immunohistochemistry

After euthanasia, the lungs were removed and fixed in 10% neutral buffered formalin overnight before embedding in paraffin. Five-micrometer sections of mouse lung tissues were cut. Some sections were stained with H&E. For immunohistochemistry, heat treatment with citrate solution (Beijing ZhongShan Golden Bridge Biotechnology Co., China) in a decloaking chamber (Biocare Medical) unmasked antigens for phosphorylated ERK (pERK), BrdU, Ki67, Endomucin and Caspase-3 staining. Whole lung tissue sections were incubated overnight at 4°C with primary antibodies: pERK (4370, Cell Signaling) at 1:100; BrdU (ab6326, Abcam) at 1:200; Ki67 (ab15580, Abcam) at 1:200; Endomucin (14-5851, eBioscience) at 1:100; cleaved Caspase-3 (9661, Cell Signaling) at 1:300. Digest-All 2B Trypsin (Invitrogen) was used to retrieve the antigen for MAC2 (CL8942AP, Cedarlane) staining at 1:5000. At 400X magnification, all MAC2-positive macrophages in tumors were counted within 3 microscope fields with the most MAC2-positive macrophages after review of the whole lung section. Three mice per genotype were analyzed.

### Proliferation analysis

At 20 weeks post-infection, mice were injected intraperitoneally with 10 μL of 10 mM BrdU in PBS per gram of body weight and euthanized after 2 hours. Whole lungs were harvested and processed as described above. At 400X magnification, all BrdU-positive tumor cell nuclei were counted within 3 microscope fields with the most BrdU-positive nuclei after review of the whole lung section. Four mice per genotype were analyzed. Same method was used to calculate Ki67-labeled tumor cells on sections from mice 28 weeks post-infection with adeno-Cre.

### Western blotting

Lung tumors were harvested from *Kras* ^*G12D*^ and *Kras* ^*G12D*^; *lL-6* ^*-/-*^ mice 32 weeks post-infection and from *Kras* ^*G12D*^; *p53* ^*flox/flox*^ and *Kras* ^*G12D*^; *p53* ^*flox/flox*^; *IL-6* ^*-/-*^ mice 15 weeks post-infection for Western blot analysis. Tumors were lysed with a homogenizer in RIPA buffer (50 mM Tris pH 7.4, 150 mM sodium chloride, 1% Nonidet P-40, 0.25% sodium deoxycholate, 1 mM EDTA) containing complete mini protease inhibitors (Roche) and phosphatase inhibitors (5870, Cell Signaling). Nuclear and Cytoplasmic Extraction Kit (CW199B, CoWin Biotech Co., Ltd. China) was used to extract cytoplasmic (C) and nuclear (N) fractions from tumors. Lysates (20 μg per lane) were separated on 10% polyacrylamide gels, transferred to PVDF filters, and incubated overnight at 4°C with antibodies to β-actin (sc-1615, Santa Cruz), pERK (4376, Cell Signaling), total-ERK (9102, Cell Signaling), pAKT (4060, Cell Signaling), total-AKT (4685, Cell Signaling), pSTAT3 (9145, Cell Signaling), STAT3 (sc-7179, Santa Cruz), p65 (sc-372, Santa Cruz), PARP (9532, Cell Signaling), GAPDH (TA-8, Beijing ZhongShan Golden Bridge Biotechnology Co., China), or β-catenin (ab32572, Abcam). Western blots were exposed to X-ray films or scanned with an ImageQuant LAS 4000mini (GE healthcare). 

### Quantitative real-time PCR

mRNA was extracted from tumors of *Kras* ^*G12D*^ and *Kras* ^*G12D*^; *lL-6* ^*-/-*^ mice 32 weeks post-infection and *Kras* ^*G12D*^; *p53* ^*flox/flox*^ and *Kras* ^*G12D*^; *p53* ^*flox/flox*^; *IL-6* ^*-/-*^ mice around 16 weeks post-infection for analysis. 2μg total RNA was reverse transcribed to cDNA using SuperRT cDNA synthesis kit (Beijing CoWin Biosciences Co., Ltd. China). Real-time PCR was performed using the BioRad iQ5 Realtime PCR system and StepOnePlus Realtime PCR system (ABI) with Realtime PCR Master Mix containing SYBR Green (QPK-201 ,TOYOBO, Japan) and unique primers (Table S1). Three to four samples for each group were detected.Gene expression results were normalized to β-actin mRNA. 

### Statistical analysis

The Student’s *t*-test was used to evaluate lesion number and number of BrdU or Ki67- positive cells. Fisher’s exact test evaluated metastatic rate. Kaplan–Meier analysis evaluated survival time. Expression differences among four groups were analyzed by ANOVA.  *P*<0.05 was considered statistically significant.

## Results

### *IL-6* deletion accelerates oncogenic *Kras* ^*G12D*^-induced lung tumorigenesis

**Figure 1:**
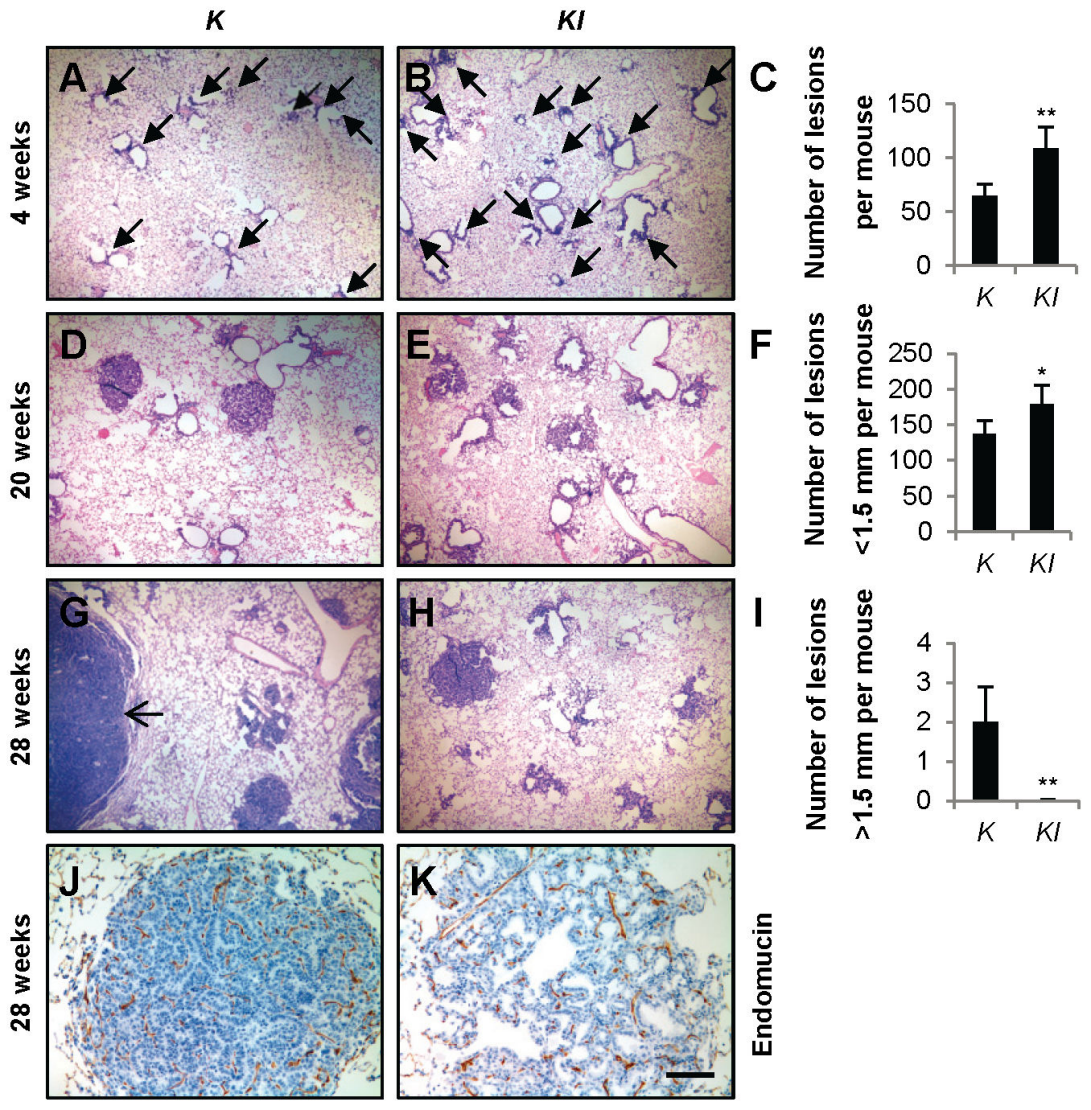
*IL-6* deletion promotes tumorigenesis but retards tumor progression of *Kras* ^*G12D*^-driven lung cancer. (A and B) Representative images of H&E-stained lung tissue sections from (A) *K* and (B) *KI* mice 4 weeks post-infection with adeno-Cre. Arrows indicate early lesions. (C) Quantification of lesions in *K* (*n*=3) and *KI* (*n*=5) mice 4 weeks post-infection with adeno-Cre. Data are shown as mean + s.e.m. **P<0.01. (D and E) Representative images of H&E-stained lung tissue sections from (D) *K* and (E) *KI* mice 20 weeks post-infection with adeno-Cre. (F) Quantification of small lesions (<1.5 mm) in *K* and *KI* mice (*n*=6) 28 weeks post-infection with adeno-Cre. Data shown are mean + s.e.m. *P<0.05. (G and H) Representative images of H&E-stained lung tissue sections from (G) *K* and (H) *KI* mice28 weeks post-infection with adeno-Cre. Arrow indicates a large tumor. (I) Quantification of large tumors (>1.5 mm) in *K* and *KI* mice (*n*=6) 28 weeks post-infection with adeno-Cre. Data shown are mean + s.e.m. **P<0.01. (J and K) Representative images of Endomucin-stained lung tissue sections from (J) *K* and (K) *KI* mice 28 weeks post-infection with adeno-Cre. Scale bar indicates 500 μm in (A, B, D, E, G and H), or 100 μm in (J and K). Abbreviations: *K*=*Kras* ^*G12D*^. *KI*=*Kras* ^*G12D*^; *IL-6* ^*-/-*^.

As previously described, *Kras* ^*G12D*^ mice developed lung tumors following a long latency [39]. *IL-6* ^-/-^ mice developed normally [38], and did not show lung tumors through 54 weeks of age (data not shown). Following adeno-Cre inhalation, PCR analysis confirmed recombination of the conditional *Kras* ^*G12D*^ allele (Figure S1). *Kras* ^*G12D*^ *; IL-6* ^*-/-*^ mice had a median survival of 37 weeks after adeno-Cre inoculation, significantly longer than *Kras* ^*G12D*^ mice (*P*<0.0001) (Table 1). Mice were euthanized at 2, 4, 20, 28, and 32 weeks post-infection, and lung lesions in H&E-stained sections were analyzed at each time point. At 2 weeks post-infection (*n* = 3), both *Kras* ^*G12D*^ and *Kras* ^*G12D*^ *; IL-6* ^*-/-*^ mice had early lung lesions. At 4 weeks post-infection, *Kras* ^*G12D*^ *; IL-6* ^*-/-*^ mice had more early lung lesions than *Kras* ^*G12D*^ mice (Figure 1A-C). These lesions were atypical adenomatous hyperplasia (AAH) and epithelial hyperplasia (EH) of the bronchioles, as reported previously [39].

**Table 1:** Comparison of lung cancer cohorts.

Genotype	Number treated	Median survival (weeks)*	Survival range (weeks)
*IL-6^-/-^*	13	>54	
*Kras^G12D^*	14	34.6	27.9 ~ 39.0
*Kras^G12D^; IL-6^-/-^*	38	37.0^a^	29.3 ~ 46.7
*p53^flox/flox^*	8	>52	
*p53^flox/flox^; IL-6^-/-^*	9	>52	
*Kras^G12D^; p53^flox/flox^*	43	16.3	11.1 ~ 19.7
*Kras^G12D^; p53^flox/flox^; IL-6^-/-^*	44	17.4^b^	12.7 ~ 23.4

^a^ *Kras* ^*G12D*^ *; IL-6* ^*-/-*^ mice had significantly longer survival than *Kras* ^*G12D*^ mice (*P*<0.0001). **^b^** *Kras* ^*G12D*^ *; p53* ^*flox/flox*^ *; IL-6* ^*-/-*^ mice had significantly longer survival than *Kras* ^*G12D*^ *; p53* ^*flox/flox*^ mice (*P*<0.01). * Median latency shown is after adeno-Cre treatment at 6-10 weeks of age, estimated by Kaplan–Meier analysis.

### *IL-6* deletion retards oncogenic *Kras* ^*G12D*^-induced lung tumor progression

At 20 weeks post-infection, lung tumors in *Kras* ^*G12D*^ *; IL-6* ^*-/-*^ mice were modestly smaller and less dense than those in *Kras* ^*G12D*^ mice (Figure 1D and E). At 28 weeks post-infection, in comparison with *Kras* ^*G12D*^ mice, more lesions were observed in *Kras* ^*G12D*^ *; IL-6* ^*-/-*^ mice with the majority of lesions in early stages of tumor development (Figure 1F). However, tumors 3-10 mm in diameter were observed in lungs of *Kras* ^*G12D*^ mice, while the majority of *Kras* ^*G12D*^ *; IL-6* ^*-/-*^ lung tumors were less than 1.5 mm (Figure 1G-I). Further, although IL-6 signaling promotes skin tumor growth and angiogenesis in a paracrine fashion [4], we did not detect any difference between *Kras* ^*G12D*^ and *Kras* ^*G12D*^ *; IL-6* ^*-/-*^ mice after immunohistochemical staining with Endomucin, a microvessel density marker to measure angiogenesis index (Figure 1J and K).

### *IL-6* deletion attenuates lung tumor proliferation

**Figure 2:**
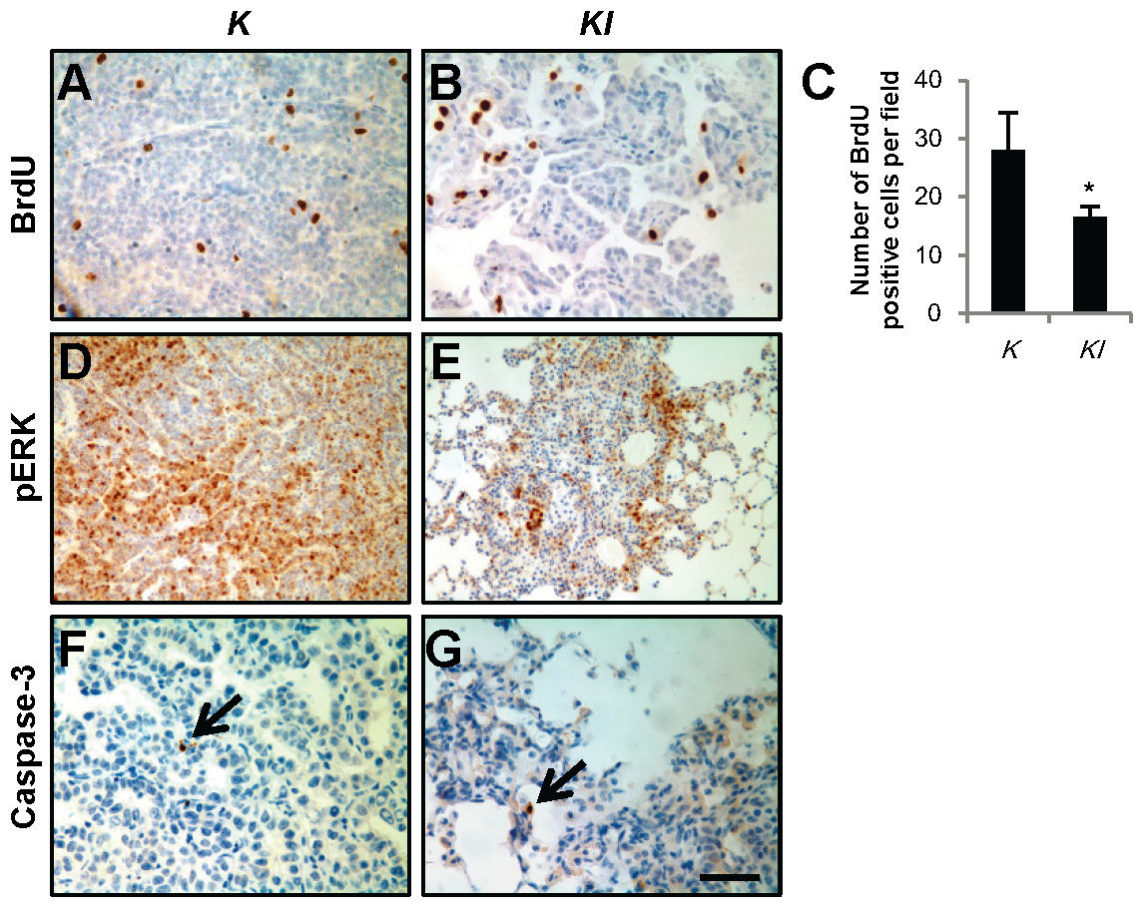
*IL-6* deletion attenuates proliferation but not apoptosis of tumor cells. (A and B) Representative images of BrdU-stained lung tissue sections from (A) *K* and (B) *KI* mice 20 weeks post-infection with adeno-Cre. (C) Quantification of BrdU-positive tumor cells in lung tissue sections of *K* and *KI* mice (*n*=4) 20 weeks post-infection with adeno-Cre. *P<0.05. (D and E) Representative images of pERK stained lung tissue sections from (D) *K* and (E) *KI* mice 20 weeks post-infection with adeno-Cre. (F and G) Representative images of cleaved Caspase-3-stained lung tissue sections from (F) *K* and (G) *KI* mice 28 weeks post-infection with adeno-Cre. Arrows indicate Caspase-3 positive tumor cells. Scale bar indicates 50 μm in (A, B, F and G), or 100 μm in (D and E) . Abbreviations: *K*=*Kras* ^*G12D*^. *KI*=*Kras* ^*G12D*^; *IL-6* ^*-/-*^.

To determine whether tumor proliferation is affected by *IL-6* deletion *in vivo*, we measured BrdU-labeling cells in lung tumors. Significantly fewer labeled nuclei were observed in lung sections from *Kras* ^*G12D*^ *; IL-6* ^*-/-*^ mice 20 weeks post-infection with adeno-Cre compared with those derived from control *Kras* ^*G12D*^ mice (Figure 2A-C). Similar results were observed from Ki67 staining in lung sections from *Kras* ^*G12D*^ and *Kras* ^*G12D*^ *; IL-6* ^*-/-*^ mice 28 weeks post-infection with adeno-Cre (Figure S2). Expression of pERK, which acts downstream of Kras and is associated with cancer cell proliferation, was reduced in tumors from *Kras* ^*G12D*^ *; IL-6* ^*-/-*^ mice 20 weeks post-infection with adeno-Cre compared to *Kras* ^*G12D*^ mice (Figure 2D and E). Caspase-3 staining revealed no differences in tumor cell apoptosis between *Kras* ^*G12D*^ and *Kras* ^*G12D*^ *; IL-6* ^*-/-*^ mice (Figure 2F and G). 

### *IL-6* deletion extends survival of *Kras* ^*G12D*^; *p53* ^*flox/flox*^ mice

As previously reported, no metastases or local invasions were detected in *Kras* ^*G12D*^ mice [39], and similar results were observed in *Kras* ^*G12D*^ *; IL-6* ^*-/-*^ mice. *Kras* ^*G12D*^ activation accompanied by *p53* deletion can cause lung tumor metastasis [37], therefore, *Kras* ^*G12D*^ *; p53* ^*flox/flox*^ *; IL-6* ^*-/-*^ mice were generated to investigate the influence of *IL-6* deletion on lung cancer metastasis.

**Figure 3:**
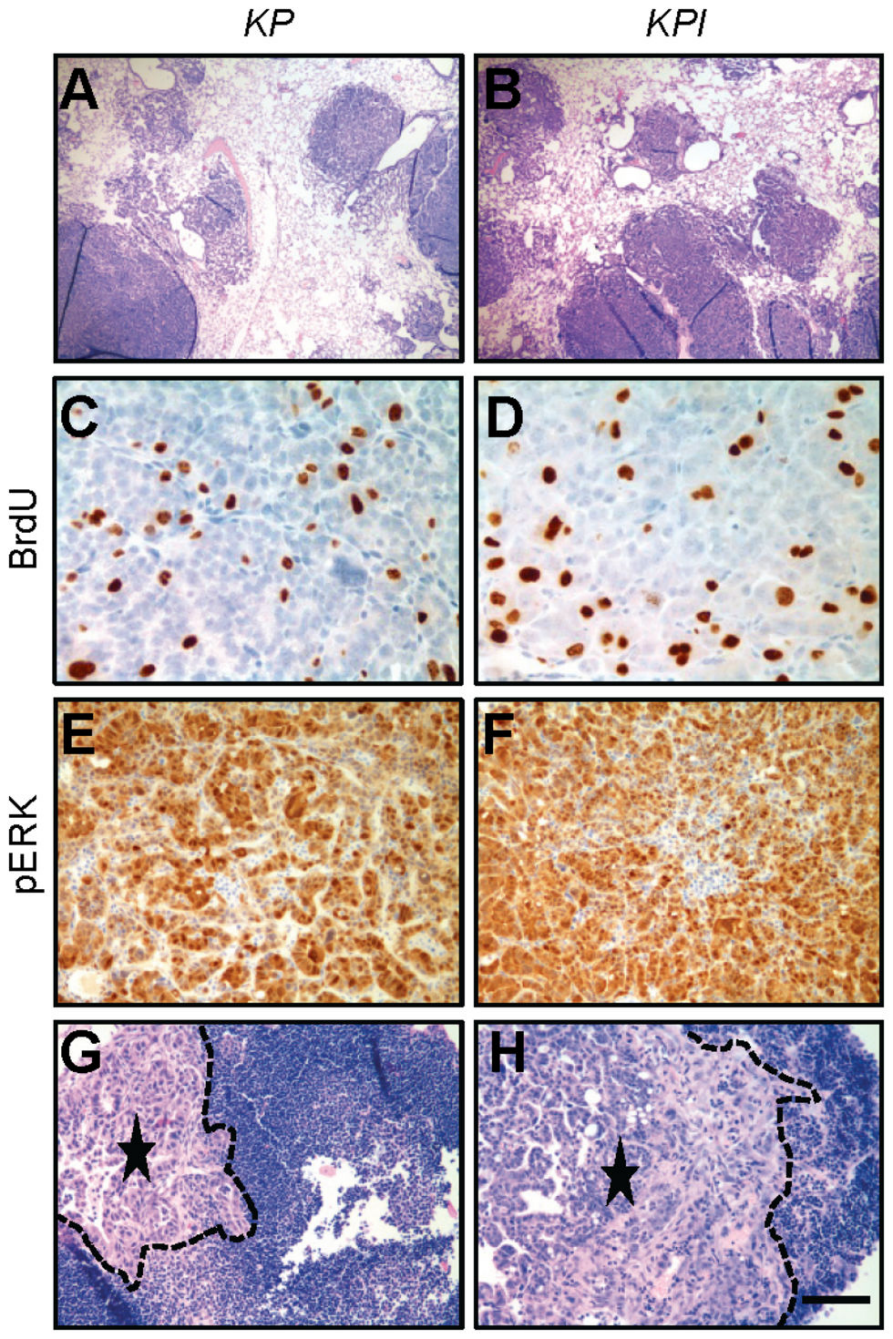
*KP* and *KPI* mice have high tumor burden, tumor cell proliferation and metastases. Representative images of lungs (A and B), BrdU staining (C and D), pERK staining (E and F) and tumor metastases (G and H) from *KP* (A, C, E, and G) and *KPI* (B, D, F, and H) mice 12 weeks post-infection with adeno-Cre. Dotted lines (G and H) show metastastic tumor edges. Asterisks indicate center of metastatic tumors. Scale bar indicates 500 μm (A, B, G, and H), 50 μm (C and D) or 100 μm (E and F). Abbreviations: *KP*=*Kras* ^*G12D*^ *; p53* ^*flox/flox*^. *KPI*=*Kras^G12D^; p53^flox/flox^*;*IL-6^-/-^*.

*p53* allelic recombination was confirmed by PCR (Figure S3). *IL-6* deletion increased median survival of *Kras* ^*G12D*^ *; p53* ^*flox/flox*^ mice (*P*<0.01) (Table 1) despite substantial lung tumor burden in both *Kras* ^*G12D*^ *; p53* ^*flox/flox*^ and *Kras* ^*G12D*^ *; p53* ^*flox/flox*^ *; IL-6* ^*-/-*^ mice 12 weeks post-infection (Figure 3A and B). BrdU staining indicated both groups of lung tumors were highly proliferative (Figure 3C and D), and pERK expression was high in both groups (Figure 3E and F); no statistical differences were observed.

For comparison, 17 *Kras* ^*G12D*^ *; p53* ^*flox/flox*^ *; IL-6* ^*-/-*^ mice and 19 *Kras* ^*G12D*^ *; p53* ^*flox/flox*^ mice were analyzed for metastases around 16 weeks post-infection with adeno-Cre (Table 2). Histologically, metastases were found in 5 of 17 *Kras* ^*G12D*^ *; p53* ^*flox/flox*^ *; IL-6* ^*-/-*^ mice (29.4%) and 10 of 19 *Kras* ^*G12D*^ *; p53* ^*flox/flox*^ mice (52.6%), although this difference was not significant (*P*=0.19). Metastatic lesions to the parietal pleura, thymus (Figure S4A and B), and lymph nodes were observed in both *Kras* ^*G12D*^ *; p53* ^*flox/flox*^ and *Kras* ^*G12D*^ *; p53* ^*flox/flox*^ *; IL-6* ^*-/-*^ mice (Figure 3G and H). Heart metastases (Figure S4C) were observed in 2 of 19 *Kras* ^*G12D*^ *; p53* ^*flox/flox*^ mice (Table 2). 

**Table 2:** Site and frequency of metastases from primary lung tumors.

Sites of metastases	*Kras^G12D^; p53^flox/flox^*	*Kras^G12D^; p53^flox/flox^; IL-6^-/-^*
Lymph node	10 of 19 (52.6%)	5 of 17 (29.4%)
Thymus	1 of 19 (5.3%)	1 of 17 (5.9%)
Heart	2 of 19 (10.5%)	0 of 17

### *IL-6* deletion alters, but *p53* deletion attenuates, some inflammatory cytokines

**Figure 4:**
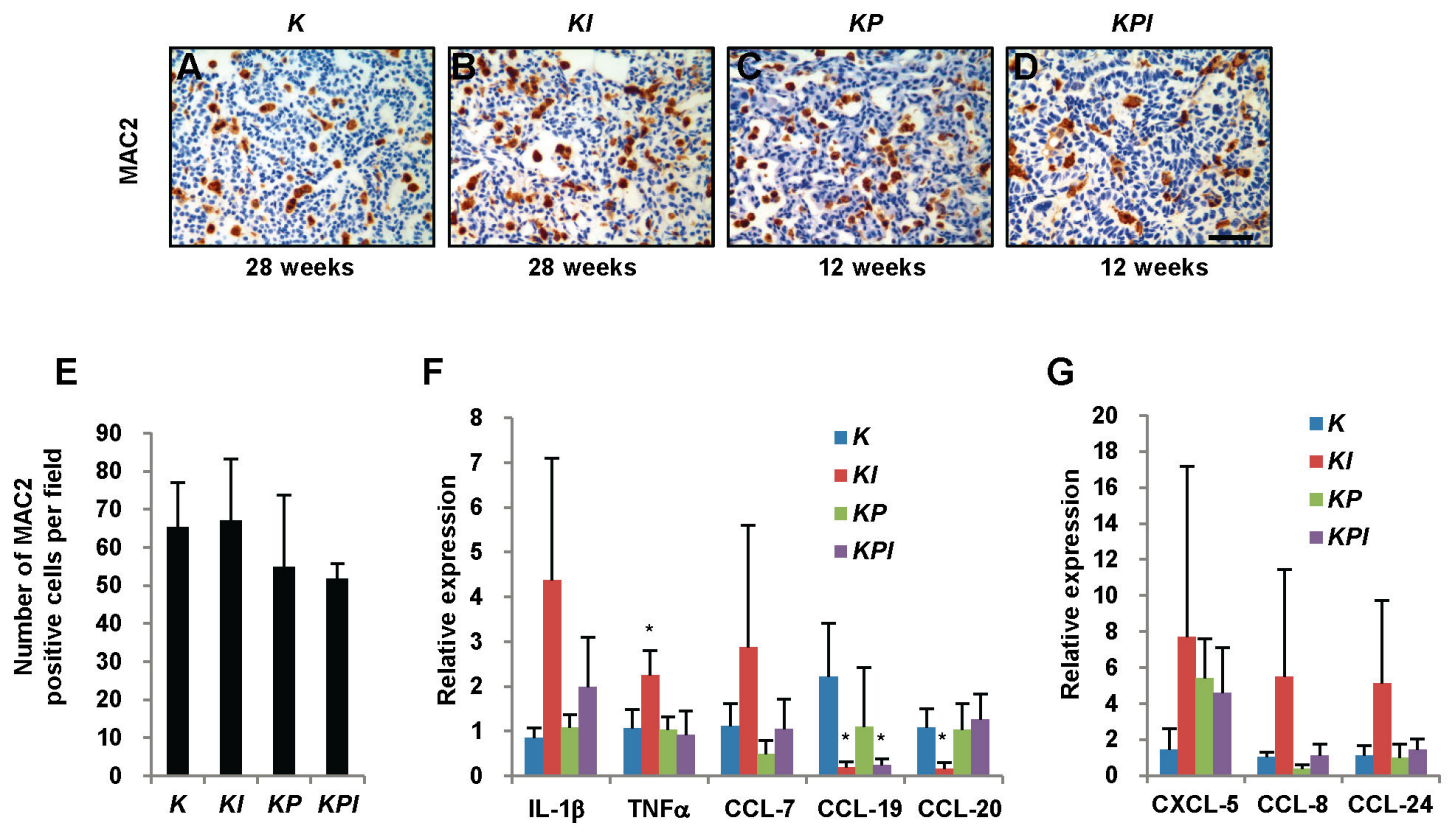
*IL-6* deletion upregulates TNFα and downregulates CCL-19 and CCL-20 in tumors. (A-D) Representative images of MAC2-stained lung tissue sections from (A) *K* and (B) *KI* mice 28 weeks post-infection and from (C) *KP* and (D) *KPI* mice 12 weeks post-infection with adeno-Cre. (E) Quantification of MAC2-positive macrophages in lung tumors from *K*, *KI, KP*, and *KPI* mice (*n*=3). No statistical difference was observed. (F and G) Gene expression of IL-1β, TNFα, CCL-7, CCL-19, CCL-20, CXCL-5, CCL-8 and CCL-24 in tumors from *K*, *KI, KP*, and *KPI* mice were determined by real-time PCR. Three to four tumors for each group were detected and triplicate PCRs were performed. Gene expression was normalized to β-actin mRNA. * *P*<0.05 vs. *K* tumors. Abbreviations: *K*=*Kras* ^*G12D*^. *KI*=*Kras* ^*G12D*^; *IL-6* ^*-/-*^. *KP*=*Kras* ^*G12D*^ *; p53* ^*flox/flox*^. *KPI*=*Kras^G12D^; p53^flox/flox^*;*IL-6* ^*-/-*^.

To investigate whether *IL-6* deletion affected inflammation, we measured macrophage density using MAC2 staining [41]. No significant changes in macrophage number were observed among tumors from *Kras* ^*G12D*^ *, Kras* ^*G12D*^ *; IL-6* ^*-/-*^ *, Kras* ^*G12D*^ *; p53* ^*flox/flox*^, and *Kras* ^*G12D*^ *; p53* ^*flox/flox*^ *; IL-6* ^*-/-*^ mice (Figure 4A-E). We also measured no change in CD3 expression, a T cell marker, in any of the four tumor groups (Figure S5B).

Several cytokines play important roles in the inflammatory process. The list includes IL-1, TNFα, and IL-6. Chemokines represent the largest family of cytokines and are classified into polypeptide groups by the location of cysteine residues near the amino terminus (e.g., C-C, C-X-C, or CX3C) [42]. Oncogenic Ras induces the secretion of the ELR1 + CXC chemokine family to promote tumorigenesis [43]. Some chemokines and growth factors are involved in tumor progression [42], so we screened inflammatory cytokine changes in tumors with real-time PCR. Three samples each tumor group were used to perform real-time PCR without replicate. There were no significant differences found for IL-1α, CXCL-1, CXCL-5, CXCL-9, CXCL-12, CXCL-16, TGF-β2, BMP2, BMP4, CCL-2, CCL-7, CCL-8, CCL-9, CCL-22, CCL-28 and CX3CL-1 expression among four groups of tumors (Figure S5). The screening results showed some changing trends in some inflammatory cytokines (Figure S5). We confirmed the changes using triplicate real-time PCR reactions with 3 to 4 samples in each group. Elevated expression of TNFα and reduced expression of CCL-19 and CCL-20 were detected in tumors from *Kras* ^*G12D*^ *; IL-6* ^*-/-*^ mice compared to *Kras* ^*G12D*^ mice. However, these changes were absent between tumors from *Kras* ^*G12D*^ *; p53* ^*flox/flox*^ and *Kras* ^*G12D*^ *; p53* ^*flox/flox*^ *; IL-6* ^*-/-*^ mice. While no statistical differences in IL-1β, CCL-7, CCL-8, CCL-24 and CXCL-5 gene expression were confirmed among four tumor groups (Figure 4F and G). 

**Figure 5:**
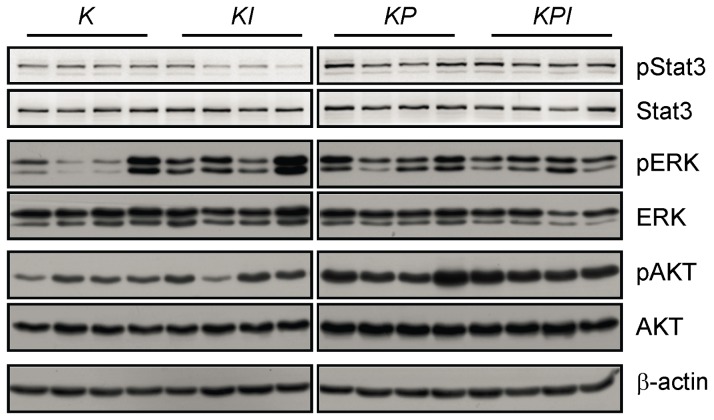
*p53* deletion Increases pSTAT3 and pAKT expression in *Kras* ^*G12D*^ tumors. Tumor lysates were extracted from *K* and *KI* mice 32 weeks post-infection and from *KP* and *KPI* mice 15 weeks post-infection for Western blot analysis. Western blot results of pSTAT3 and total-STAT3 were scanned by an ImageQuant LAS 4000mini (GE healthcare). Other results were exposed to X-ray films. Abbreviations: *K*=*Kras* ^*G12D*^. *KI*=*Kras* ^*G12D*^; *IL-6* ^*-/-*^. *KP*=*Kras* ^*G12D*^ *; p53* ^*flox/flox*^. *KPI*=*Kras^G12D^; p53^flox/flox^*;*IL-6* ^*-/-*^.

We also examined the nuclear localization of NF-κB subunit p65, which is important in cancer-related inflammation and malignant progression [44,45]. However, no significant localization change was observed among tumors from the four genotypes (Figure S6). And no dramatic change was observed in β-catenin expression in nucleus (Figure S6), which is related to lung cancer development [46]. Expression of pSTAT3, which is the main downstream target of IL-6, was reduced in some *Kras* ^*G12D*^ *; IL-6* ^*-/-*^ tumors (Figure 5) but increased in *p53-*deleted tumors. These data indicated that *IL-6* deletion altered tumor expression of some inflammatory cytokines, although these changes were weakened by *p53* deletion.

## Discussion

Previous studies have shown that carcinogen-induced tumorigenesis in *IL-6* ^*−/−*^ mice is delayed by 1-2 weeks [4,47]; however, we found no difference in *Kras* ^*G12D*^-induced tumor onset regardless of *IL-6* deficiency. One possible explanation is that *Kras* ^*G12D*^ activation may induce lung tumorigenesis more robustly than other carcinogens. 

Some inflammatory cytokines are associated with tumor progression [42]. TNFα may act as a tumor promoter by regulating a cascade of cytokines, chemokines, adhesions, matrix metalloproteinases (MMPs) and pro-angiogenic activities [2,48]. In this study, *IL-6* deletion in *Kras* ^*G12D*^ tumors upregulated TNFα expression. Elevated expression of TNFα may compensate for the loss of IL-6 and thus increase tumorigenesis. However, tumor progression is delayed in *Kras* ^*G12D*^ *; IL-6* ^*-/-*^ mice, consistent with previous results [4,47]. These data indicate that *IL-6* is important for tumor progression *in vivo* and suggest that IL-6 inhibition may have biphasic stage-specific effects in lung cancer, enhancing tumorigenesis early while suppressing tumor progression later. Consequently, this may pose a risk to lung cancer patients treated with IL-6-targeted therapy. 

CCL-20 (or macrophage pro-inflammatory chemokine-3α, MIP-3α), a C-C motif chemokine, is overexpressed in pancreatic carcinoma cells and stimulates growth of tumor cells [49]. CCL-19 (or macrophage inflammatory protein-3 beta, MIP-3β), plays an important role in the migration of mature dendritic cells and T-cells [50]. Both dendritic cells and T-cells are double-edged swords in the tumor microenvironment, in addition to initiating potent anti-tumor immune responses, these cells may also stimulate cancerous cell growth and spreading [51,52]. Persistently activated or tyrosine-phosphorylated STAT3 (pSTAT3) is found in 50% of lung adenocarcinomas [53,54]. pSTAT3 can enhance tumor proliferation and loss of pSTAT3 arrests growth of premalignant lesions, almost abrogating the development of advanced tumors [55]. In this study, *IL-6* deletion in *Kras* ^*G12D*^ tumors resulted in downregulation of pSTAT3, CCL-19 and CCL-20. pERK expression was reduced in *Kras* ^*G12D*^ *; IL-6* ^*-/-*^ tumors 20 weeks post-infection (Figure 2), but increased in most *Kras* ^*G12D*^ *; IL-6* ^*-/-*^ tumors 32 weeks post-infection (Figure 5). These data suggest that early stage, tumor growth may be delayed by low expression of pERK, pSTAT3 and CCL-20. During later stages, tumor growth may be induced by upregulation of pERK and TNFα, although these mechanisms need further study.

Our data show that *p53* deletion more dramatically affected *Kras* ^*G12D*^-induced lung cancer than *IL-6* deletion. To a large extent, *p53* deletion attenuated the effects of *IL-6* deletion on delayed tumor growth and prolonged survival. *p53* deletion enhanced pSTAT3 expression (Figure 5) and abrogated the change in CCL-20 expression in *Kras* ^*G12D*^ *; p53* ^*flox/flox*^ *; IL-6* ^*-/-*^ tumors (Figure 4). *p53* deletion also increased expression of pAKT and total-AKT expression, which are associated with high proliferation, in *Kras* ^*G12D*^ *; p53* ^*flox/flox*^ and *Kras* ^*G12D*^ *; p53* ^*flox/flox*^ *; IL-6* ^*-/-*^ tumors(Figure 5). *p53* deletion may attenuate the effects of *IL-6* deletion through these pathways.

We observed a trend for reduced metastases with *IL-6* deletion (Table 2), although additional samples are required to confirm this result. Separately, we have observed dramatically increased IL-6 expression in primary and metastatic tumors from mice with high metastatic rates (unpublished data), similar to the report that IL-6 promotes cancer cells to metastasize to distant sites [30,31]. Furthermore, survival time of *Kras* ^*G12D*^ *; p53* ^*flox/flox*^ *; IL-6* ^*-/-*^ mice was significantly extended (*P*< 0.01) (Table 1). These results indicate that *IL-6* deletion may reduce lung cancer metastases and prolong survival time *in vivo* although *p53* deletion dominantly impacts the evolution of *Kras* ^*G12D*^ lung cancer.

The involvement of inflammation in tumorigenesis, progression, and metastasis is widely accepted; however, whether IL-6-targeted therapies will prolong the survival time of lung cancer patients remains uncertain. Our results indicate anti-IL-6 therapies may have some success in clinical trials. For example, when NSCLC has not metastasized, IL-6 inhibition may prolong survival but increase the risk of further tumorigenesis; if metastasized, IL-6 inhibition may only moderately impact metastasis but may lengthen survival time. Further studies are needed to elucidate these possibilities. In summary, our results provide evidence that *IL-6* deficiency promotes lung tumorigenesis, but suppresses tumor progression and elongates survival *in vivo*. However, these effects can be attenuated by *p53* deletion.

## Supporting Information

Figure S1 **PCR analysis of *Kras* allelic recombination.** A 500 bp PCR product represents the floxed, unrecombined *Kras* ^G12D^ allele; a 622 bp fragment represents the wildtype *Kras* allele; and a 650 bp fragment represents a recombined *Kras* ^*G12D*^ allele after removal of floxed stop cassette by adeno-Cre. *K*, KI, KP, and *KPI* mice were treated with adeno-Cre and the 650 bp recombined band revealed. Abbreviations: *WT*= wildtype lungs. Floxed=floxed *Kras* ^*G12D*^, without adeno-Cre treatment. *K*=*Kras* ^*G12D*^. *KI*=*Kras* ^*G12D*^; IL-6^-/-^. *KP*=*Kras* ^*G12D*^ *; p53* ^*flox/flox*^. *KPI*=*Kras^G12D^; p53^flox/flox^*;*IL-6* ^*-/-*^.
(TIF)

Figure S2 ***IL-6* deletion attenuates tumor proliferation determined by Ki67 staining.** (A and B) Representative images of Ki67-stained lung tissue sections from (A) *K* and (B) *KI* mice 28 weeks post-infection with adeno-Cre. (C) Quantification of Ki67-positive tumor cells in lung tissue sections of *K* and *KI* mice (*n*=3). **P<0.01. Scale bar indicates 50 μm. Abbreviations: *K*=*Kras* ^*G12D*^ *. KI*=*Kras* ^*G12D*^; *IL-6* ^*-/-*^.
(TIF)

Figure S3 **PCR analysis of *p53* allelic recombination.** A 212 bp PCR product represents the floxed, unrecombined *p53* allele; a 168 bp fragment represents the recombined allele after inoculation with adeno-Cre; and a 130 bp fragment represents the wildtype *p53* allele. *K*, *KI, KP*, and *KPI* mice were treated with adeno-Cre. The 168 bp recombined band was showed in *KP* and *KPI* mice and 212 bp fragment remained due to tumor stromal cells. Abbreviations: *WT*=wildtype lungs*. Floxed*=floxed *p53*, without adeno-Cre treatment. *K*=*Kras* ^*G12D*^ *. KI*=*Kras* ^*G12D*^; *IL-6* ^*-/-*^ *. KP*=*Kras* ^*G12D*^ *; p53* ^*flox/flox*^ *. KPI*=*Kras^G12D^; p53^flox/flox^*;*IL-6* ^*-/-*^.
(TIFF)

Figure S4 ***KP* and *KPI* mice develop metastatic lesions.** (A and B) Representative images of metastatic lesions to the (A) pleura, (B) thymus in *KPI* mice 15 weeks post-infection with adeno-Cre. Dotted lines in the images indicate metastatic tumor edges. Asterisks indicate center of metastatic tumors. (C) Representative image of heart metastases in *KP* mice 14 weeks post-infection with adeno-Cre. Metastatic lesions in the heart are left of the dotted line. Scale bar indicates 200 μm (A) or 100 μm (B and C). Abbreviations: *KP*=*Kras* ^*G12D*^ *; p53* ^*flox/flox*^ *. KPI*=*Kras^G12D^; p53^flox/flox^*;*IL-6* ^*-/-*^.
(TIF)

Figure S5 **Real-time PCR screen of changes in inflammatory cytokines levels.** .
Three tumors from each genotype were analyzed by real-time PCR without replicate for expression of the indicated cytokine. Gene expression was normalized to β-actin mRNA. *P<0.05 vs. *K* tumors. # *P*<0.05 vs. *KP* tumors. Abbreviations: *K*=*Kras* ^*G12D*^. *KI*=*Kras* ^*G12D*^; *IL-6* ^*-/-*^. *KP*=*Kras* ^*G12D*^ *; p53* ^*flox/flox*^ *. KPI*=*Kras^G12D^; p53^flox/flox^*;*IL-6* ^*-/-*^.
(TIF)

Figure S6 **Nuclear localization of p65 and β-catenin are unchanged.** Tumors from each mouse genotype were lysed to obtain cytoplasmic (C) and nuclear (N) fractions. Lysates were analyzed for the presence of nuclear p65 and β-catenin by Western blot. Fraction purity was determined by GAPDH (cytoplasmic) and PARP (nuclear) blots. Abbreviations: *K*=*Kras* ^*G12D*^ *. KI*=*Kras* ^*G12D*^; *IL-6* ^*-/-*^ *. KP*=*Kras* ^*G12D*^ *; p53* ^*flox/flox*^ *. KPI*=*Kras^G12D^; p53^flox/flox^*;*IL-6* ^*-/-*^.
(TIFF)

Table S1 **Primers for real-time PCR analysis of gene expression.**
(DOCX)
