# Supplementary figures and images for: Loss of p53 Attenuates the Contribution of IL-6 Deletion on Suppressed Tumor Progression and Extended Survival in Kras-Driven Murine Lung Cancer
Source: PLoS One. 2013 Nov 15;8(11):e80885. doi: 10.1371/journal.pone.0080885 (PMC3829911; doi:10.1371/journal.pone.0080885)

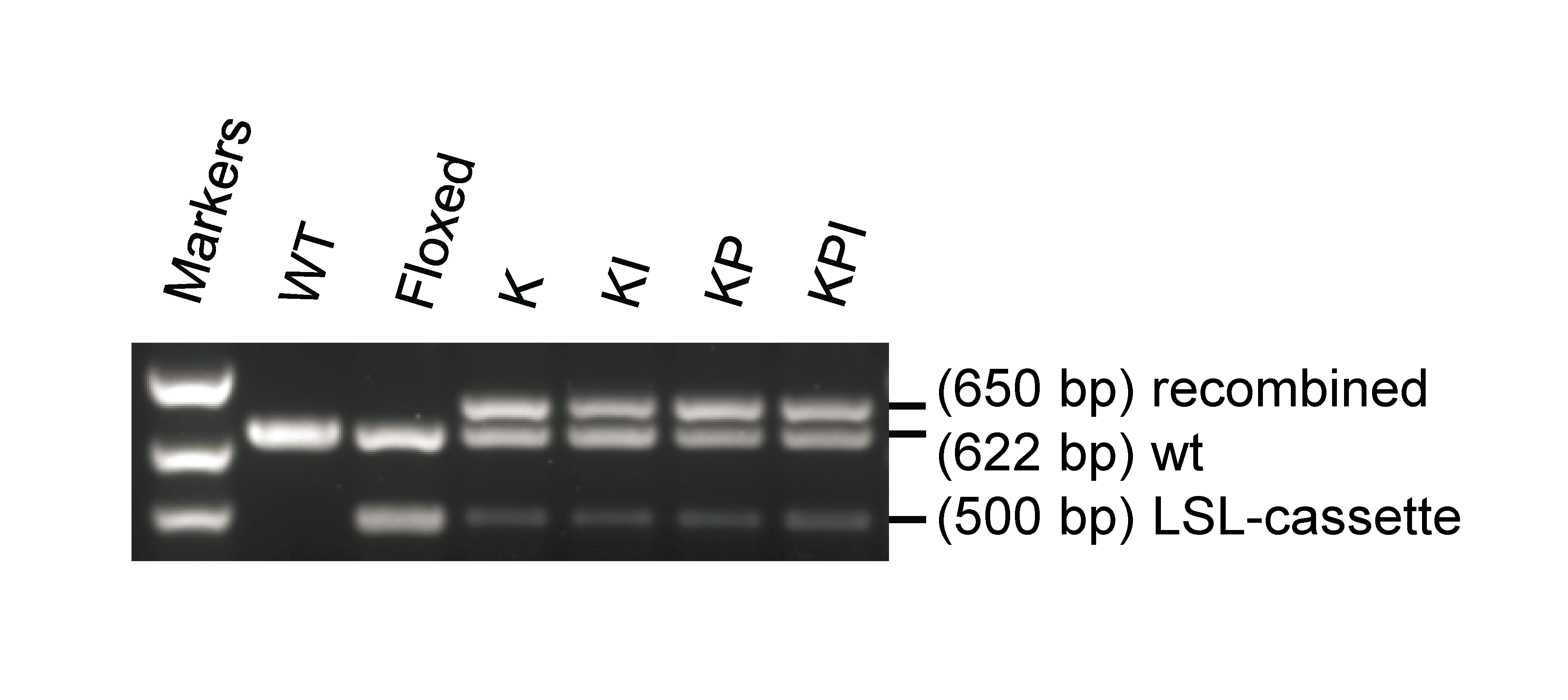

Supplement: Figure S1 — PCR analysis of Kras allelic recombination. A 500 bp PCR product represents the floxed, unrecombined Kras G12D allele; a 622 bp fragment represents the wildtype Kras allele; and a 650 bp fragment represents a recombined Kras G12D allele after removal of floxed stop cassette by adeno-Cre. K, KI, KP, and KPI mice were treated with adeno-Cre and the 650 bp recombined band revealed. Abbreviations: WT= wildtype lungs. Floxed=floxed Kras G12D, without adeno-Cre treatment. K=Kras G12D. KI=Kras G12D; IL-6-/-. KP=Kras G12D ; p53 flox/flox. KPI=KrasG12D; p53flox/flox;IL-6 -/-. (TIF) [file pone.0080885.s001.tif]

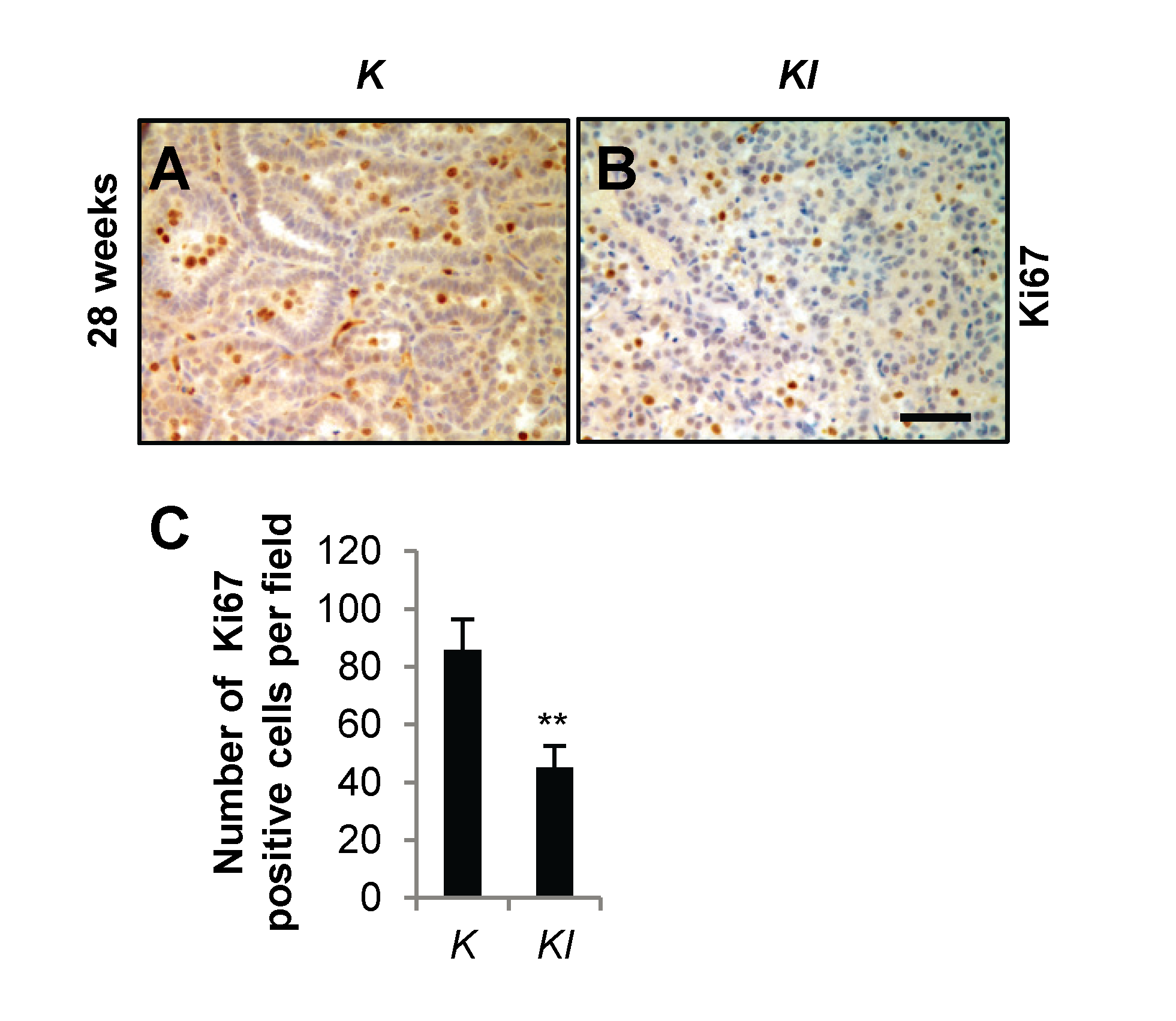

Supplement: Figure S2 — IL-6 deletion attenuates tumor proliferation determined by Ki67 staining. (A and B) Representative images of Ki67-stained lung tissue sections from (A) K and (B) KI mice 28 weeks post-infection with adeno-Cre. (C) Quantification of Ki67-positive tumor cells in lung tissue sections of K and KI mice (n=3). **P<0.01. Scale bar indicates 50 μm. Abbreviations: K=Kras G12D . KI=Kras G12D; IL-6 -/-. (TIF) [file pone.0080885.s002.tif]

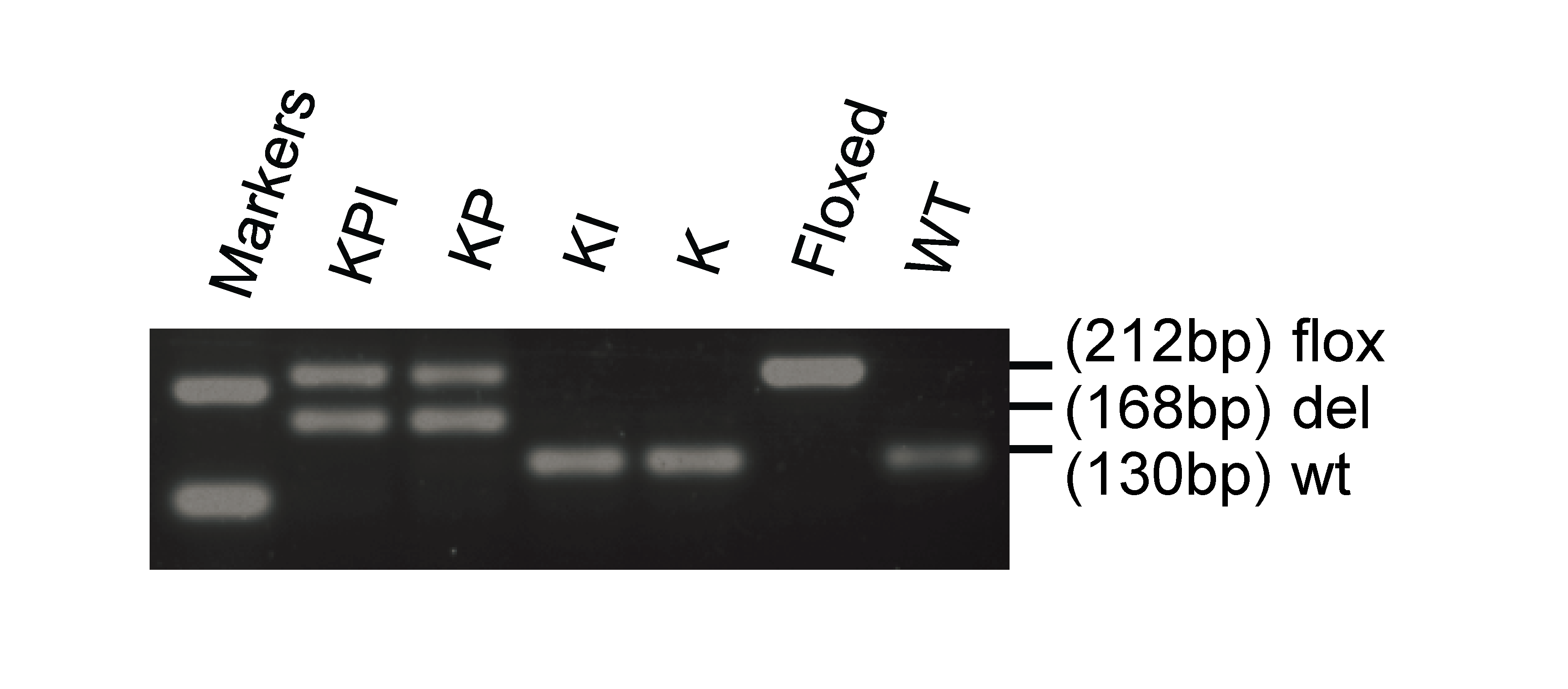

Supplement: Figure S3 — PCR analysis of p53 allelic recombination. A 212 bp PCR product represents the floxed, unrecombined p53 allele; a 168 bp fragment represents the recombined allele after inoculation with adeno-Cre; and a 130 bp fragment represents the wildtype p53 allele. K, KI, KP, and KPI mice were treated with adeno-Cre. The 168 bp recombined band was showed in KP and KPI mice and 212 bp fragment remained due to tumor stromal cells. Abbreviations: WT=wildtype lungs. Floxed=floxed p53, without adeno-Cre treatment. K=Kras G12D . KI=Kras G12D; IL-6 -/- . KP=Kras G12D ; p53 flox/flox . KPI=KrasG12D; p53flox/flox;IL-6 -/-. (TIFF) [file pone.0080885.s003.tiff]

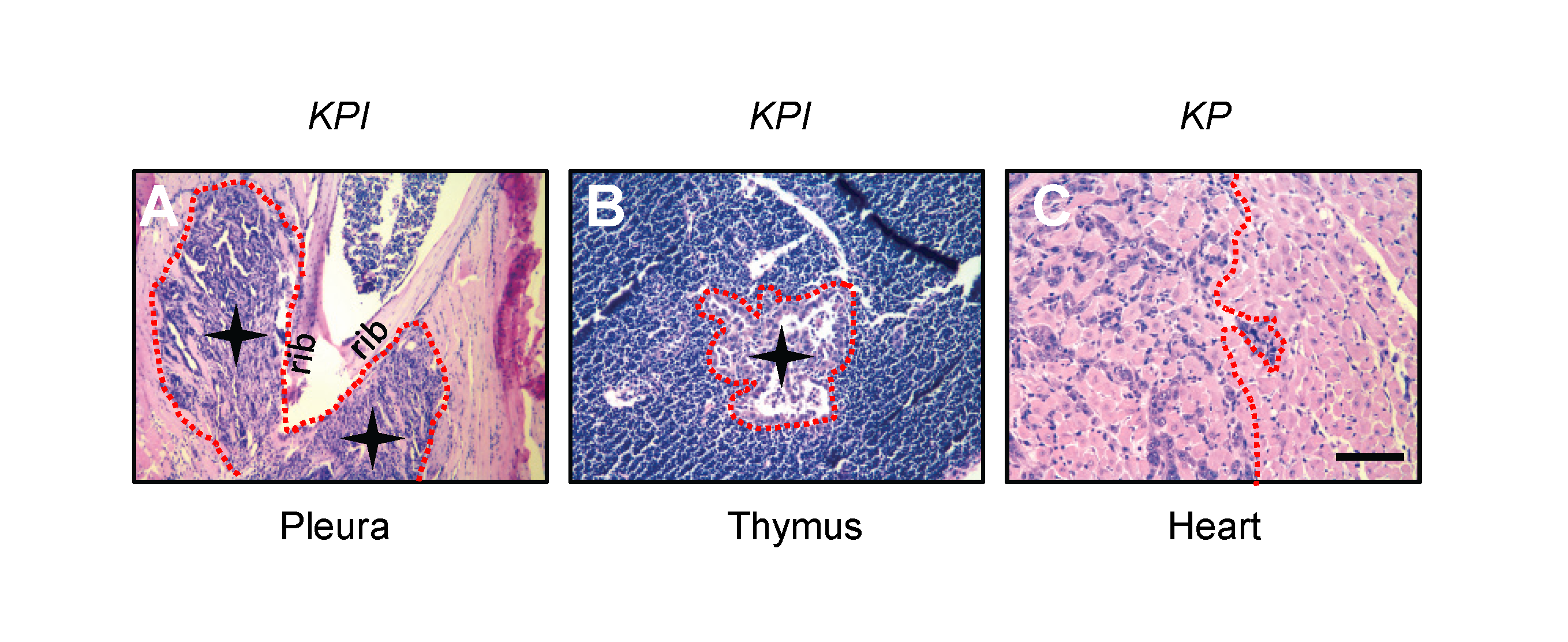

Supplement: Figure S4 — KP and KPI mice develop metastatic lesions. (A and B) Representative images of metastatic lesions to the (A) pleura, (B) thymus in KPI mice 15 weeks post-infection with adeno-Cre. Dotted lines in the images indicate metastatic tumor edges. Asterisks indicate center of metastatic tumors. (C) Representative image of heart metastases in KP mice 14 weeks post-infection with adeno-Cre. Metastatic lesions in the heart are left of the dotted line. Scale bar indicates 200 μm (A) or 100 μm (B and C). Abbreviations: KP=Kras G12D ; p53 flox/flox . KPI=KrasG12D; p53flox/flox;IL-6 -/-. (TIF) [file pone.0080885.s004.tif]

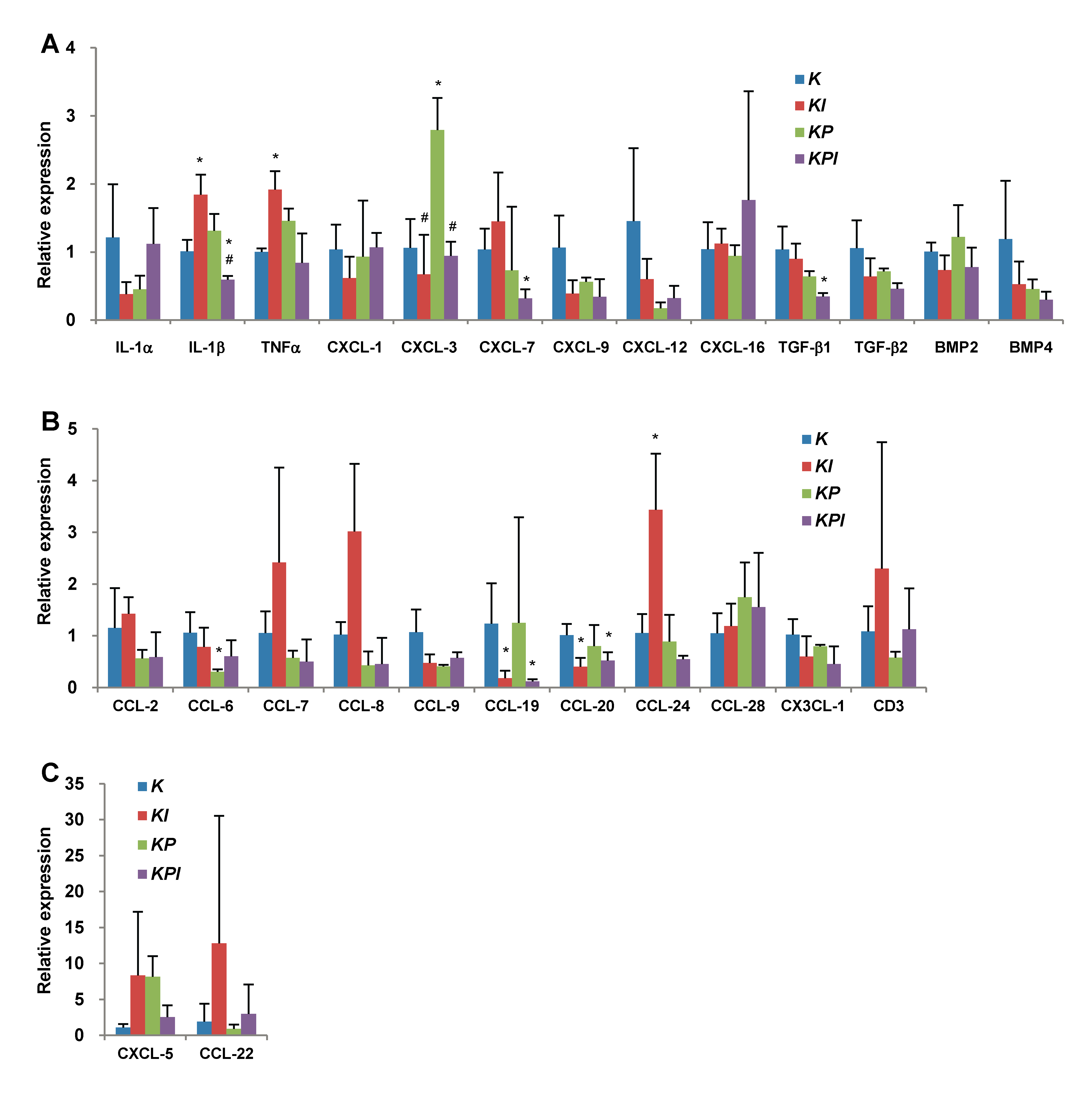

Supplement: Figure S5 — Real-time PCR screen of changes in inflammatory cytokines levels. . Three tumors from each genotype were analyzed by real-time PCR without replicate for expression of the indicated cytokine. Gene expression was normalized to β-actin mRNA. *P<0.05 vs. K tumors. # P<0.05 vs. KP tumors. Abbreviations: K=Kras G12D. KI=Kras G12D; IL-6 -/-. KP=Kras G12D ; p53 flox/flox . KPI=KrasG12D; p53flox/flox;IL-6 -/-. (TIF) [file pone.0080885.s005.tif]

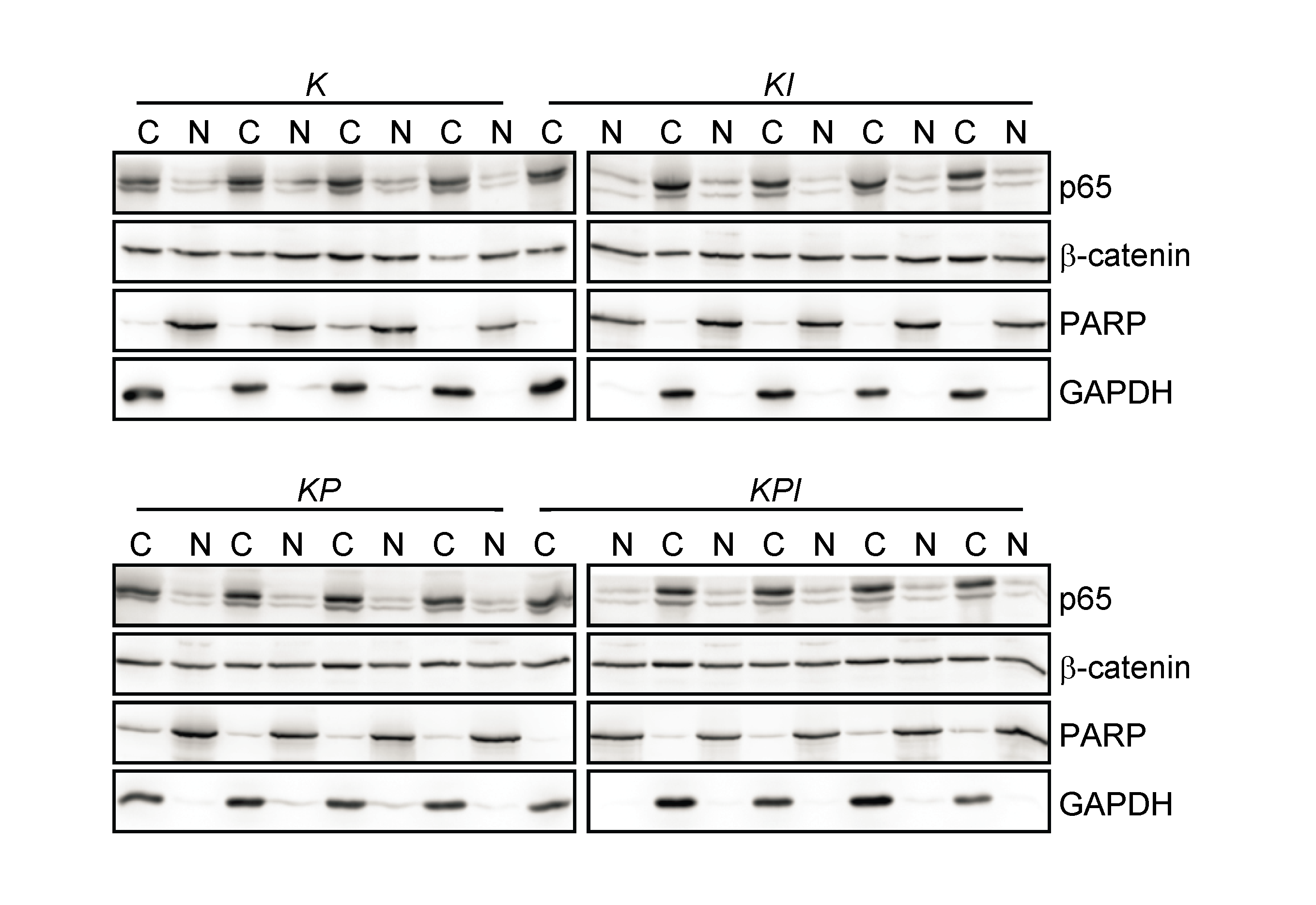

Supplement: Figure S6 — Nuclear localization of p65 and β-catenin are unchanged. Tumors from each mouse genotype were lysed to obtain cytoplasmic (C) and nuclear (N) fractions. Lysates were analyzed for the presence of nuclear p65 and β-catenin by Western blot. Fraction purity was determined by GAPDH (cytoplasmic) and PARP (nuclear) blots. Abbreviations: K=Kras G12D . KI=Kras G12D; IL-6 -/- . KP=Kras G12D ; p53 flox/flox . KPI=KrasG12D; p53flox/flox;IL-6 -/-. (TIFF) [file pone.0080885.s006.tiff]
