# Supplementary material for: Loss of p53 Attenuates the Contribution of IL-6 Deletion on Suppressed Tumor Progression and Extended Survival in Kras-Driven Murine Lung Cancer
Source: PLoS One. 2013 Nov 15;8(11):e80885. doi: 10.1371/journal.pone.0080885 (PMC3829911; doi:10.1371/journal.pone.0080885)
Supplement: Table S1 — Primers for real-time PCR analysis of gene expression. (DOCX) [file pone.0080885.s007.docx]

**Table S1. Primers for real-time PCR analysis of gene expression.**

| IL-1α | S | CCCGTGTTGCTGAAGGAGTTG |
| --- | --- | --- |
|  | AS | GCACCCGACTTTGTTCTTTGG |
|  |  |  |
| IL-1β | S | TGTCTTTCCCGTGGACCTTCC |
|  | AS | CCGACAGCACGAGGCTTTTT |
|  |  |  |
| TNFα | S | GCCCAGACCCTCACACTCAGA |
|  | AS | CTTTGAGATCCATGCCGTTGG |
|  |  |  |
| CXCL-1 | S | CAGACCATGGCTGGGATTCAC |
|  | AS | TCGCGACCATTCTTGAGTGTG |
|  |  |  |
| CXCL-3 | S | CACCAACCACCAGGCTACAGG |
|  | AS | CGTCACCGTCAAGCTCTGGAT |
|  |  |  |
| CXCL-5 | S | CACAGTGCCCTACGGTGGAAG |
|  | AS | ACTGCGAGTGCATTCCGCTTA |
|  |  |  |
| CXCL-7 | S | GGCCTGCCCACTTCATAACCT |
|  | AS | CATCTGCAGCGCAGTTCGATA |
|  |  |  |
| CXCL-9 | S | ATAAGGAATGCACGATGC |
|  | AS | TCTTCACATTTGCCGAGT |
|  |  |  |
| CXCL-12 | S | CATTGACCCGAAATTAAA |
|  | AS | CTCTTCTTCTGTCGCTTCT |
|  |  |  |
| CXCL-16 | S | CGCAGGGTACTTTGGATC |
|  | AS | CTCGTGTCCGAAGGTGTC |
|  |  |  |
| CCL-2 | S | ATGCAGTTAACGCCCCACTCA |
|  | AS | ACCCATTCCTTCTTGGGGTCA |
|  |  |  |
| CCL-6 | S | TGTGGCTGTCCTTGGGTC |
|  | AS | CTCCTGCTGATAAAGATGAT |
|  |  |  |
| CCL-7 | S | CCCCAAGAGGAATCTCAA |
|  | AS | ACAGCTTCCCAGGGACACC |
|  |  |  |
| CCL-8 | S | AAGGCTCCAGTCACCTGC |
|  | AS | ACCCTGCTTGGTCTGGAAAA |
|  |  |  |
| CCL-9 | S | AGATTGCTGCCTGTCCT |
|  | AS | TAGGTCCGTGGTTGTGA |
|  |  |  |
| CCL-19 | S | CCTTCCGCTACCTTCTTAA |
|  | AS | TGTTGCCTTTGTTCTTGG |
|  |  |  |
| CCL-20 | S | ACAGACGCCTCTTCCTTC |
|  | AS | TCACCCAGTTCTGCTTTG |
|  |  |  |
| CCL-22 | S | ACTACATCCGTCACCCTC |
|  | AS | AGTAGCTTCTTCACCCAGA |
|  |  |  |
| CCL-24 | S | TCTTGCTGCACGTCCTTT |
|  | AS | TATGGCCCTTCTTGGTGA |
|  |  |  |
| CCL-28 | S | CTGACGGGGACTGCGACCT |
|  | AS | ACGATTGTGCGGGCTGAT |
|  |  |  |
| CX3CL-1 | S | TCCGCTATCAGCTAAACCA |
|  | AS | TCCACCCGCTTCTCAAAC |
|  |  |  |
| TGFβ1 | S | ACCCCCACTGATACGCCTGA |
|  | AS | GCAGTGAGCGCTGAATCGAA |
|  |  |  |
| TGFβ2 | S | CCTTCGTGCCGTCTAATA |
|  | AS | GGTCTTCCCACTGGTTTT |
|  |  |  |
| BMP2 | S | CTCAATGGACGTGCCCCCTA |
|  | AS | CCTGGGGAAGCAGCAACACT |
|  |  |  |
| BMP4 | S | GGGAACCGGGCTTGAGTACC |
|  | AS | TCCACCTGCTCCCGAAAGAG |
|  |  |  |
| CD3 | S | TCTTGCTAGGACCGCTTAT |
|  | AS | CTGGCTGTGCTTTCTGTG |
|  |  |  |
| β-actin | S | GCTTCTTTGCAGCTCCTTCGT |
|  | AS | GACCCATTCCCACCATCACA |
